# *Phyllanthus emblica* Fruit Improves Obesity by Reducing Appetite and Enhancing Mucosal Homeostasis via the Gut Microbiota–Brain–Liver Axis in HFD-Induced Leptin-Resistant Rats

**DOI:** 10.1021/acs.jafc.4c01226

**Published:** 2024-04-25

**Authors:** Hsin-Yu Chang, Sheng-Yi Chen, Jer-An Lin, Ying-Yin Chen, Ying-Ying Chen, Yu-Chen Liu, Gow-Chin Yen

**Affiliations:** † Department of Food Science and Biotechnology, National Chung Hsing University, 145 Xingda Road, Taichung 40227, Taiwan; ‡ Graduate Institute of Food Safety, National Chung Hsing University, 145 Xingda Road, Taichung 40227, Taiwan

**Keywords:** Phyllanthus emblica fruit, obesity, leptin resistance, microbiota–gut–brain–liver axis, intestinal mucosal barrier, tight junction protein, appetite-related neuropeptides, antimicrobial peptides

## Abstract

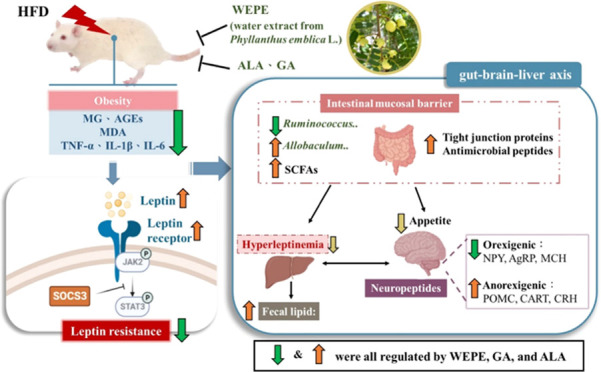

The impact of leptin resistance on intestinal mucosal barrier integrity, appetite regulation, and hepatic lipid metabolism through the microbiota–gut–brain–liver axis has yet to be determined. Water extract of *Phyllanthus emblica* L. fruit (WEPE) and its bioactive compound gallic acid (GA) effectively alleviated methylglyoxal (MG)-triggered leptin resistance *in vitro*. Therefore, this study investigated how WEPE and GA intervention relieve leptin resistance-associated dysfunction in the intestinal mucosa, appetite, and lipid accumulation through the microbiota–gut–brain–liver axis in high-fat diet (HFD)-fed rats. The results showed that WEPE and GA significantly reduced tissues (jejunum, brain, and liver) MG-evoked leptin resistance, malondialdehyde (MDA), proinflammatory cytokines, SOCS3, orexigenic neuropeptides, and lipid accumulation through increasing leptin receptor, tight junction proteins, antimicrobial peptides, anorexigenic neuropeptides, excretion of fecal triglyceride (TG), and short-chain fatty acids (SCFAs) via a positive correlation with the *Allobaculum* and *Bifidobacterium* microbiota. These novel findings suggest that WEPE holds the potential as a functional food ingredient for alleviating obesity and its complications.

## 1 Introduction

It is predicted that by 2035, more than 50% of individuals worldwide will be overweight and obese.^1^ Obesity causes leptin resistance, which reduces the ability of leptin to suppress appetite. Accordingly, the vicious cycle of physical inactivity associated with obesity-caused leptin resistance exacerbates a large population of individuals with obesity and disease-associated burdens. Reports predict that the global cost of overweight and obesity will reach 4.32 trillion dollars annually in 2035,^1^ indicating that a significant human health issue should be resolved. Although pharmacotherapy for obesity is approved by the FDA,^2^ side effects such as headache, gastrointestinal disorders, palpitations, tachycardia, anxiety, and hypertension are observed.^3^ Thus, discovering a natural food for attenuating obesity is a valuable strategy for reducing costs and side effects.

*Phyllanthus emblica* L., generally known as Indian gooseberry, is an edible plant belonging to the Phyllanthaceae family that contains abundant bioactive compounds such as gallic acid (GA), ellagic acid (EA), vitamin C, minerals, β-glucogallin (β-glu), and tannins.^4^ Our previous research demonstrated that the water extract of *P. emblica* fruit (WEPE) and GA effectively improve methylglyoxal (MG)-derived insulin resistance, oxidative stress, inflammation, and cognitive decline through inhibiting RAGE/MAPK/NF-κB signaling and restoring gut microbiota disorders in high-fat diet (HFD)-administered rats.^5^ Additionally, we first revealed that MG-glycated leptin caused leptin resistance, which exacerbated lipid accumulation by downregulating lipid metabolism-related genes (PPAR-α/CPT-1) and upregulating genes associated with lipogenesis signaling cascade (SREBP-1C/ACC/FAS) expression. These effects can be counteracted by treatment with WEPE (150 μg/mL) and GA (1.49 μM) in HepG2 cells subjected to free fatty acid (FFA) incubation,^6^ indicating that *P. emblica* L. is an excellent natural food for eliminating MG-induced leptin resistance, oxidative stress, inflammation, and cognitive decline in individuals with obesity.

Leptin plays an essential role in suppressing appetite and increasing energy expenditure through downregulating agouti-related protein (AgRP) and upregulating pro-opiomelanocortin (POMC).^7^ In addition, AgRP-produced neuropeptide Y (NPY) is connected with increased food intake and decreased energy expenditure.^8^ Additionally, chronic hyperleptinemia lifts the coexpression of NPY and cocaine- and amphetamine-regulated transcripts (CARTs) to increase food intake and reduce energy expenditure.^9^ Similarly, exogenous leptin administration promotes energy metabolism and thermoregulation by regulating corticotropin-releasing hormone (CRH) in *Eothenomys miletus*.^10^ Furthermore, a clinical report showed that 2 days of fasting significantly increases circulating melanin-concentrating hormone (MCH) levels in combination with a decrease in leptin levels in a cross-sectional study of 108 healthy subjects.^11^ The above evidence indicates that attenuation of leptin resistance is strongly correlated to reducing appetite and enhancing energy expenditure.

Microbial diversity and composition in individuals with obesity affect host lipid deposition, insulin and leptin resistance, inflammation, oxidative stress, and energy homeostasis.^12^ Moreover, several reports revealed that the gut–brain axis modulates appetite and food intake.^13,14^ For example, a leptin sensitizer containing a 9-amino-acid peptide named D3 can enhance the presence of intestinal *Akkermansia muciniphila* and upregulate the expression of uroguanylin, subsequently inhibiting lipid absorption and appetite by targeting the hypothalamus in HFD-fed specific pathogen-free (SPF) mice.^15^

In addition, leptin not only is a critical mediator of appetite regulation but also induces intestinal epithelial cells to express antimicrobial peptides for mucosal homeostasis,^16^ indicating the possibility of leptin regulating appetite through the gut–brain axis. However, the antimicrobial peptides involved in appetite regulation via the leptin-modulated gut–brain axis have not been thoroughly investigated. Furthermore, our previous studies revealed that both WEPE and GA effectively trap MG and subsequently suppress leptin resistance, lipid accumulation, inflammation, oxidative stress, and dysbiosis in HFD-fed rats,^5,6^ indicating that WEPE and GA may influence appetite-related gene expression through the leptin-modulated gut–brain axis. Although leptin is strongly associated with regulating multiple appetite-related genes in hypothalamic neurons, the intracellular mechanisms underlying the modulation of this process by the gut–brain–liver axis and leptin have not been fully elucidated. Thus, this study aimed to investigate the impact of WEPE and GA on regulating the expression of appetite- and antimicrobial-peptide-associated genes via the leptin-modulated gut–brain axis in HFD-administered rats.

## 2 Materials and Methods

### 2.1 Materials

Bovine serum albumin (BSA), gallic acid (GA), butanol, 1,1,3,3-tetramethoxypropane, *o*-phenylenediamine (OPD), 2-methylquinoxaline (2-MQ), 5-methylquinoxaline, sodium hydroxide (NaOH), sodium nitrate (NaNO_3_), triglyceride quantification kit, total bile acids (TBA) assay kit, propionic acid, 2-nitrophenylhydrazine (2-NPH), and Folin–Ciocalteu reagent were bought from Sigma-Aldrich (St. Louis, MO). Alagebrium chloride (ALA) and *N*-(3-(dimethylamino)propyl)-*N*-ethylcarbodiimide hydrochloride were bought from Tokyo Chemical Industry (Tokyo, Japan). Tumor necrosis factor-α (TNF-α, DY510), Interleukin-1β (IL-1β, DY501), and Interleukin-6 (IL-6, DY506-05) enzyme-linked immunosorbent assay (ELISA) kits were bought from R&D System (Minneapolis, Minnesota). Glacial acetic acid, hydrochloric acid (HCl), and methanol were bought from ECHO CHEMICAL Co., Ltd., Miaoli, Taiwan. TOOLS easy fast RT kit and TOOLS 2xSYBR qPCR mix were obtained from BIOTOOLS Co., Ltd., New Taipei City, Taiwan. Phosphatase inhibitor cocktail and protease inhibitor cocktail were purchased from MedChemExpress (Monmouth Junction, NJ). Bicinchoninic acid (BCA) protein assay kit was bought from BD Biosciences (Franklin Lakes, New Jersey). Tetramethylbenzidine substrate was bought from Clinical Science Products (Mansfield, MA). Sodium carbonate (Na_2_CO_3_) and sulfuric acid (H_2_SO_4_) were obtained from UNION CHEMICAL WORKS Ltd., Taichung, Taiwan. Beef tallow was purchased from Dacheng suppliers (Taichung, Taiwan). Acetonitrile, trichloroacetic acid (TCA), and perchloric acid were bought from Thermo Fisher Scientific (Waltham, MA). Thiobarbituric acid (TBA) was bought from MERCK (Darmstadt, Hesse, Germany). RNA extraction reagent was bought from EBL Biotechnology (Taipei, Taiwan). Chloroform and isopropanol were obtained from Avantor (Radnor, PA). Cholesterol CHOD PAP kit was purchased from Fortress Diagnostics (Antrim, Ulster, U.K.). Pyridine was bought from TEDIA (Phoenix, AZ). Potassium hydroxide (KOH) was bought from Shimakyu’s Pure Chemicals (Osaka, Japan). Butyric acid was bought from Alfa Aesar (Haverhill, MA). Laboratory rodent diet (LabDiet 5001) was purchased from Newco Distributors Corporation (Rancho Cucamonga, CA). Primer was bought from Genomics (Taipei, Taiwan).

### 2.2 Extraction of *P. emblica* Fruit

The fruits of *P. emblica* L. were supplied by the Miaoli District Agricultural Research and Extension Station, Council of Agriculture, Executive Yuan (Miaoli, Taiwan). The fruits were dried in hot air at 60 °C and powdered using a high-speed grinder (RT-08, Rong Tsong, Taichung, Taiwan). The powder was then soaked in deionized water and stirred overnight. The extract was subsequently filtered and lyophilized to obtain the water extract of *P. emblica* fruit (WEPE). The WEPE was stored at −80 °C for further experiments.^5^

### 2.3 Determination of Total Phenolics and Flavonoids

The total phenolic and flavonoid contents were determined following the protocol described in a previous report.^17^ Briefly, WEPE was mixed with the Folin–Ciocalteu reagent and incubated in the dark at room temperature for 1 h. Subsequently, the absorbance of the mixture was determined using a 750 nm spectrophotometer (BMG Labtech, Ortenberg, Germany). The total phenolic content was expressed as gallic acid equivalents (GAE), while the flavonoid content was expressed as catechin equivalents (CE).

### 2.4 Determination of Polyphenolic Compounds and β-Glucogallin

The evaluation of major polyphenolic compounds and β-glucogallin content in WEPE was conducted according to previous methods.^5^ The WEPE solution was mixed with ddH_2_O and then filtered through a 0.22 μm filter membrane before analysis. The high-performance liquid chromatography (HPLC) system used for analysis consisted of the following instruments: Chromaster 5110 Pump, Chromaster 5210 Auto Sample, Chromaster 5310 Column Oven, and Chromaster 5430 Diode Array Detector (all from Hitachi, Tokyo, Japan).

For the analysis, a LiChrospher 100 RP-18 Column (5 μm, 4 mm × 250 mm) (Merck KGaA, Darmstadt, Germany) was used. The mobile phase consisted of two components: A was 2% (v/v) acetic acid in water, and B was a mixture of 0.5% (v/v) acetic acid in water and acetonitrile (50:50, v/v). The gradient conditions were as follows: B increases from 5 to 10% from 0 to 10 min, increases to 25% from 10 to 40 min, increases to 50% from 40 to 60 min, increases to 70% from 60 to 65 min, increases to 100% from 65 to 70 min, decreases to 50% from 70 to 75 min, decreases to 30% from 75 to 80 min, and decreases to 2% from 80 to 82 min. The UV–vis detector detects the absorbance wavelength at 254 nm. The injection volume for the sample was 20 μL, and the flow rate was set at 0.8 mL/min. The column temperature was maintained at 40 °C during the analysis.

Additionally, the analysis of β-glucogallin was performed using different conditions on the same HPLC system. The mobile phase for this analysis consisted of A, which was 1% (v/v) formic acid in water, and B, which was 1% (v/v) formic acid. The gradient conditions were as follows: B was held at 2% from 0 to 10 min, increased to 37% from 10 to 37 min, held at 37% from 37 to 42 min, increased to 40% from 42 to 60 min, increased to 60% from 60 to 70 min, increased to 100% from 70 to 90 min, held at 100% from 90 to 104 min, decreased to 2% from 104 to 105 min, and held at 2% from 105 to 112 min. The UV–vis detector measured the absorption at a wavelength of 310 nm. The injection volume for the sample was 3 μL, and the flow rate was set at 0.8 mL/min. The column temperature was controlled at 35 °C during the analysis.

### 2.5 Experimental Animals

Five-week-old male Sprague–Dawley (SD) rats were obtained from the BioLASCO Experimental Animal Center (Taipei, Taiwan). The rats were fed with LabDiet 5001 and housed in a room with controlled environmental conditions, including a temperature of 22 ± 2 °C, humidity of 65 ± 5%, and a 12-h light–dark cycle. Following a 1-week adaptation period, the rats were randomly divided into six groups (*n* = 5/per group): the control group (LabDiet 5001), HFD group (LabDiet 5001 + 40% beef tallow, providing a total of 60% kcal), L-WEPE group (LabDiet 5001 + 40% beef tallow + low-dose WEPE 250 mg/kg b.w.), H-WEPE group (LabDiet 5001 + 40% beef tallow + high-dose WEPE 500 mg/kg b.w.), ALA group (LabDiet 5001 + 40% beef tallow + alagebrium chloride 1 mg/kg b.w.), and GA group (LabDiet 5001 + 40% beef tallow + GA 100 mg/kg b.w.).

Except for those in the control group, all of the rats were fed HFD for 112 consecutive days, and the treatments were administered daily via intragastric gavage. Afterward, the rats were anesthetized using 3% isoflurane and sacrificed. Samples, including liver, brain, jejunum, perirenal fat, epididymal fat, mesenteric fat, brain tissues, and blood, were collected for further analysis. All procedures were carried out in accordance with the guidelines approved by the Institutional Animal Care & Use Committee of the National Chung Hsing University (Approval No. 108-95^R^), and the study followed the principles outlined in the Guide for the Care and Use of Laboratory Animals (eighth edition).

### 2.6 Hematoxylin–Eosin (H&E) Staining

Hematoxylin–eosin (H&E) staining was performed on liver and perirenal adipose tissue slices. Staining and subsequent injury scoring were carried out by Professor Jiunn-Wang Liao (Department of Veterinary Medicine, National Chung Hsing University). The H&E-stained tissues were assessed and scored on a scale from 1 to 5 based on the extent of lesion damage, following the scoring criteria of previous reports.^18^

### 2.7 Serum Biochemical Analysis

After isoflurane anesthesia was administered, blood samples were collected from the rats via an orbital route and transferred to serum separation tubes. The tubes were then centrifuged at 2000 rpm for 10 min to obtain the serum. The high-density lipoprotein cholesterol (HDL-C), low-density lipoprotein cholesterol (LDL-C), triglyceride (TG), total cholesterol (TC), aspartate aminotransferase (AST), alanine aminotransferase (ALT), blood urea nitrogen (BUN), and creatinine (CRE) levels in the serum were measured by the Union Clinical Laboratory (Taichung, Taiwan) using an ADVIA Chemistry XPT System (Siemens, Munich, Germany).

### 2.8 Analysis of Proinflammatory Cytokines

To extract proteins, small intestine, brain, and liver tissues were treated with lysis buffer and homogenized using a tissue homogenizer (SH-100, Kurabo, Osaka, Japan) at a speed of 800 rpm until complete homogenization was achieved for each tissue sample. The protein concentration in the homogenized samples was determined using a BCA protein assay kit following the instructions provided in the manual.

ELISA kits were used to measure the TNF-α, IL-1β, and IL-6 levels. Initially, the primary antibody was used to precoat a 96-well plate that was left overnight. After the plate was washed, the tissue homogenate was added to the wells and allowed to react at room temperature for 1 h. Following three washes, the secondary antibody was added, and the mixture was incubated at room temperature for 2 h. The plate was then washed three additional times, and streptavidin-HRP was added to facilitate a reaction in the dark at room temperature for 20 min. To stop the reaction, 1 N H_2_SO_4_ (stop solution) was added, and the levels of proinflammatory cytokines were measured at an absorbance of 450 nm.

### 2.9 Analysis of Malondialdehyde (MDA) Content

The tissue homogenate was combined with the TCA-TBA-HCl reagent and subjected to heating at 100 °C. Next, butanol was added to terminate the reaction. The resulting pink supernatant was quantified by using an ELISA spectrophotometer (BMG Labtech, Ortenberg, Germany). The content of MDA in the tissues was determined by calculation using 1,1,3,3-tetramethoxypropane as a standard.

### 2.10 Measurement of Methylglyoxal (MG)

MG was derivatized to 2-methylquinoxaline (2-MQ) using the OPD reaction for measurement via HPLC.^5^ Briefly, the process involved incubating the OPD with a tissue homogenate in the dark at 37 °C for 24 h to convert MG to 2-MQ. After derivatization, the internal standard 5-MQ was added, and the mixture was subjected to solid-phase extraction (SPE) using an InertSep Pharma column (GL Sciences, Tokyo, Japan). The resulting mixture was then filtered through a 0.22 μm membrane and analyzed for 2-MQ content using HPLC.

The HPLC system used for analysis consisted of a Chromaster 5110 pump, a Chromaster 5210 autosampler, a Chromaster 5310 column oven, and a Chromaster 5430 diode array detector (all from Hitachi, Tokyo, Japan). The analytical column used was a LiChrospher 100 RP-18 column (5 μm, 4 × 250 mm) from Merck KGaA (Merck KGaA, Darmstadt, Germany). The mobile phase consisted of 5 mM NaH_2_PO_4_ in water (A) and acetonitrile (B). The gradient program for elution was as follows: 0–21 min, 17.6% B; 21–22 min, 50% B; 22–23 min, 50% B; 23–24 min, 17.6% B; and 24–35 min, 17.6% B. The sample injection volume was 20 μL, and the flow rate was 1 mL/min. The column temperature was maintained at 30 °C, and the absorbance at 317 nm was monitored via a UV–vis detector.

### 2.11 Measurement of Tissue AGE Content

To analyze the advanced glycation end product (AGE) contents in tissues, a FLUO star galaxy spectrophotometer from BMG Labtech (Offenburg, Germany) was used to detect the autofluorescence of AGEs at 405 and 355 nm in a 96-well black plate.^6^ Briefly, the tissues were homogenized with NaOH, and then a 0.2 M boric acid solution was added to the supernatant until the pH reached 8.5. The absorbance of the supernatants obtained from feces or brain tissue was measured by using a spectrophotometer, and the relative fluorescence intensity of the AGEs was calibrated against that of a native bovine serum albumin (BSA) solution. The fluorescence intensity of the native BSA solution was defined as 1 arbitrary unit (AU).

### 2.12 Gene Expression Analysis

Real-time polymerase chain reaction (PCR) was conducted according to previous methods.^6^ Briefly, tissues were homogenized using the TRIzol reagent. Total RNA was purified, and cDNA was synthesized using a TOOLS easy fast RT kit. Real-time PCR was performed in a StepOneTM real-time PCR system (Applied Biosystems, Foster City, CA) following the below conditions: initial denaturation (95 °C, 15 min), denaturation (95 °C, 10 s), annealing (50–61 °C, 20 s), and extension (72 °C, 45 s). The primer sequences and annealing temperatures used are provided in Table S1. After the analysis, the relative gene expression was calculated by determining the cycle threshold (CT) using the 2^–ΔΔCT^ method and normalizing the expression to that of the internal reference gene (housekeeping gene).

### 2.13 Analysis of Fecal Lipid Content

The fecal fat content was analyzed following previous methods using the chloroform/methanol method.^19^ Briefly, 100 mg of dried fecal powder was mixed with 1 mL of chloroform/methanol (2:1, v/v) and 200 μL of ddH_2_O. This mixture allowed for the extraction of lipids from the fecal sample. The mixture was centrifuged at 13,000 rpm for 10 min, after which the organic phase at the bottom was collected. The dried fecal crude fat was then dissolved in 100% isopropanol to obtain fecal fat extracts for subsequent analysis of fecal triglyceride, cholesterol, and bile acid levels. The contents of fecal lipids were measured using a triglyceride quantification kit, cholesterol CHOD PAP kit, and total bile acid (TBA) assay kit according to the instruction manual.

### 2.14 Analysis of Short-Chain Fatty Acids (SCFAs)

The SCFA content was assessed according to our previous report.^18^ Briefly, the feces were incubated with 70% ethanol, after which the supernatant was mixed with derivatization reagents and extracted with ether. The filtered SCFA hydrazide solution was subjected to HPLC analysis (Hitachi, Tokyo, Japan).

### 2.15 Analysis of the Gut Microbiota

Gut microbiota analysis was performed according to previous methods.^20^ Fecal samples were collected, and genomic DNA was extracted using an AllPure Genomic DNA Extraction Kit from Allbio (Allbio, Taichung, Taiwan). The concentration of the extracted DNA was determined using a Qubit 2.0 fluorometer from Invitrogen (Castle Beach, CA). Next-generation sequencing (NGS) was performed using 16S rDNA variable regions V3–V4, and taxonomic analysis was subsequently conducted. The forward primer sequence “CTAGGRRBGCASCAGKVRVGAAT” and the reverse primer sequence “GGACTACNVGGGTWTCTAATCC” were used to amplify the V3 and V4 regions by PCR. DNA library preparation, sequencing (Silva_132), and Spearman correlation analysis were performed by AllBio Science, Inc. (Taichung, Taiwan).

### 2.16 Statistical Analysis

Significant differences between groups were determined by one-way analysis of variance (ANOVA) and Duncan’s multiple range test using Statistical Package for Science (SPSS) version 25 (SPSS Software, Chicago, IL). *p* < 0.05 (mean ± SEM) was considered to indicate statistical significance.

## 3 Results

### 3.1 Bioactive Compounds in the WEPE

In this study, the WEPE exhibited high levels of total polyphenols (139.5 ± 2.3 mg of gallic acid equivalents per gram of extract) and total flavonoids (24.7 ± 0.3 mg of catechin equivalents per gram of extract). Furthermore, the phenolic compound composition of WEPE was determined using HPLC, and the concentrations of specific compounds were calculated based on standard calibration curves. The major phenolic components identified in WEPE included GA (2.4 ± 0.03 mg/g of extract), EA (3.2 ± 0.05 mg/g of extract), and β-glucogallin (3.8 ± 0.1 mg/g of extract).

### 3.2 WEPE Ameliorated Obesity Symptoms by Suppressing Obesity-Associated Oxidative Stress and Inflammation in HFD-Fed Rats

**Figure 1:**
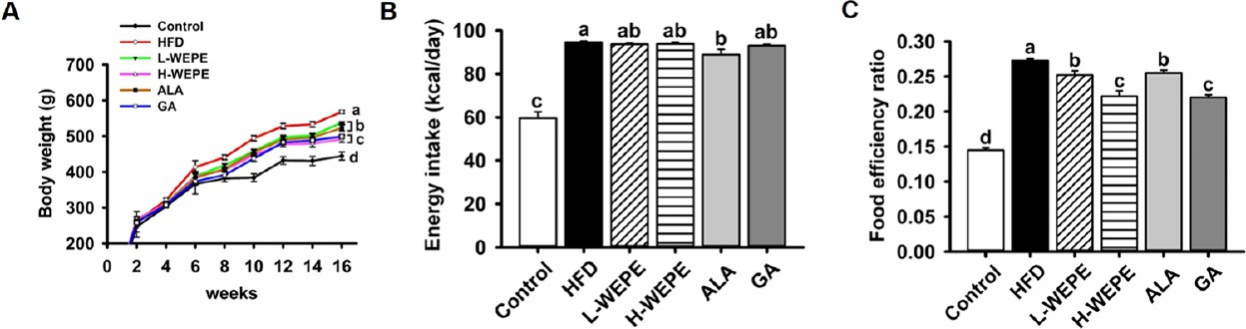
Effect of WEPE administration on (A) body weight, (B) energy intake, and (C) the food efficiency ratio in HFD-induced SD rats. Values with different letters in each column are significantly different (*p* < 0.05, *n* = 5).

**Figure 2:**
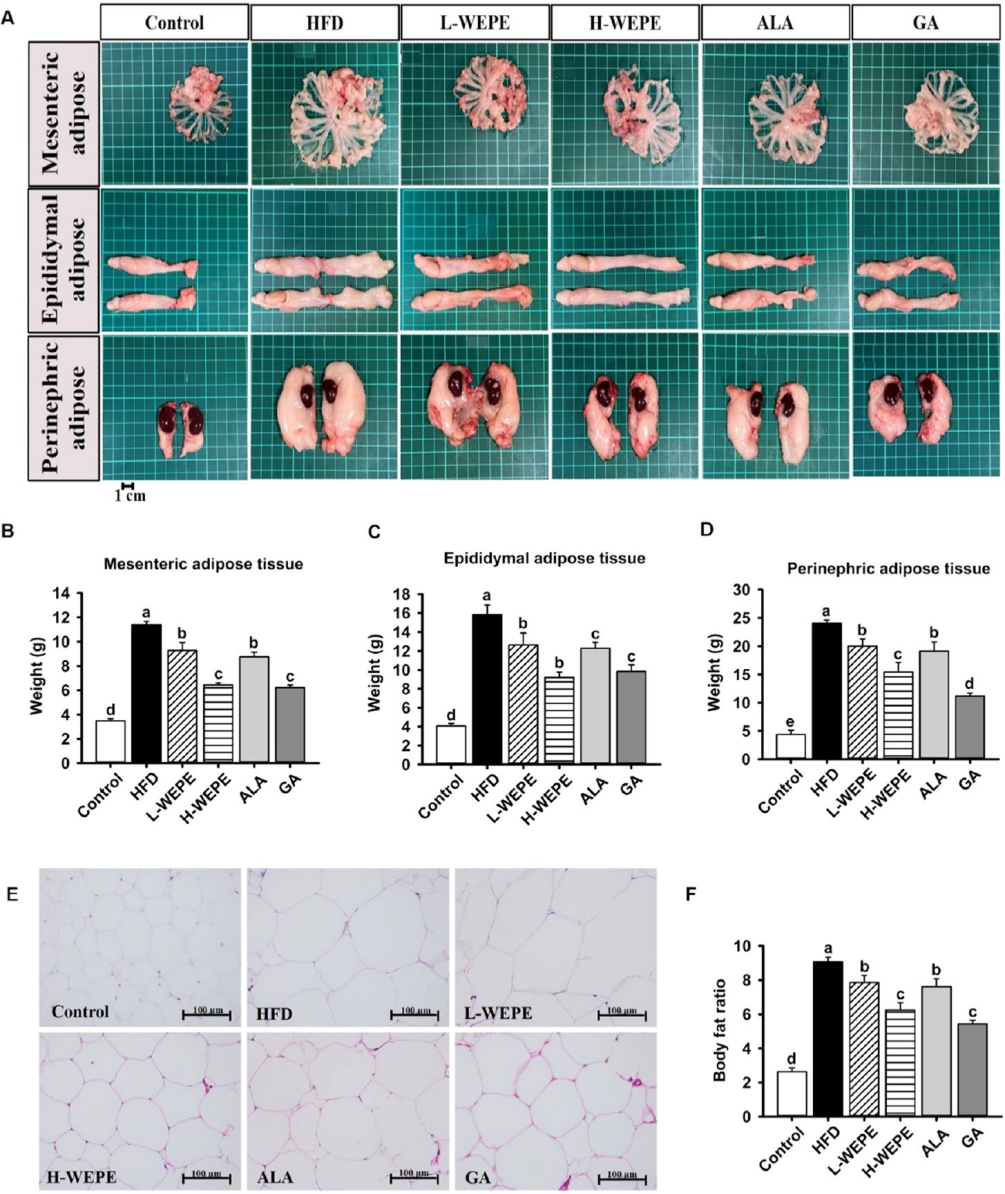
Effect of WEPE administration on the (A) appearance of mesenteric, epididymal, and perinephric adipose tissues, (B) weight of mesenteric adipose tissue, (C) weight of epididymal adipose tissue, (D) weight of perinephric adipose tissue, (E) size of adipose tissue, and (F) body fat ratio (%). The calculation of the body fat ratio is the body fat mass (mesenteric adipose tissue + epididymal adipose tissue + perinephric adipose tissue)/body weight × 100. Values with different letters in each column are significantly different (*p* < 0.05, *n* = 5).

Body weight and energy intake were dramatically greater in the HFD-fed group than in the control group (Figure 1A,B). WEPE, ALA, and GA supplementation effectively reversed weight gain in HFD-fed rats (Figure 1A). Similarly, the food efficiency ratio was significantly greater in the HFD group than in the control group but was lower in L-WEPE, H-WEPE, ALA, and GA groups (Figure 1C). As expected, hypertrophy of adipose tissue was clearly observed in the mesenteric, epididymal, and perinephric tissues, which was suppressed by WEPE, ALA, and GA treatments (Figure 2A). The quantified results showed that the weights of mesenteric, epididymal, and perinephric tissues were strongly increased in the HFD group, which were all improved in HFD-fed rats subjected to WEPE, ALA, or GA administration, respectively (Figure 2B–D). For example, the hypertrophy of epididymal adipose tissue in the HFD group was distinct from that in the other groups (Figure 2E). Moreover, the highest body fat ratio was detected in the HFD group, which was not only dose-dependently reduced by WEPE treatment but also inhibited by ALA and GA treatment (Figure 2F).

**Figure 3:**
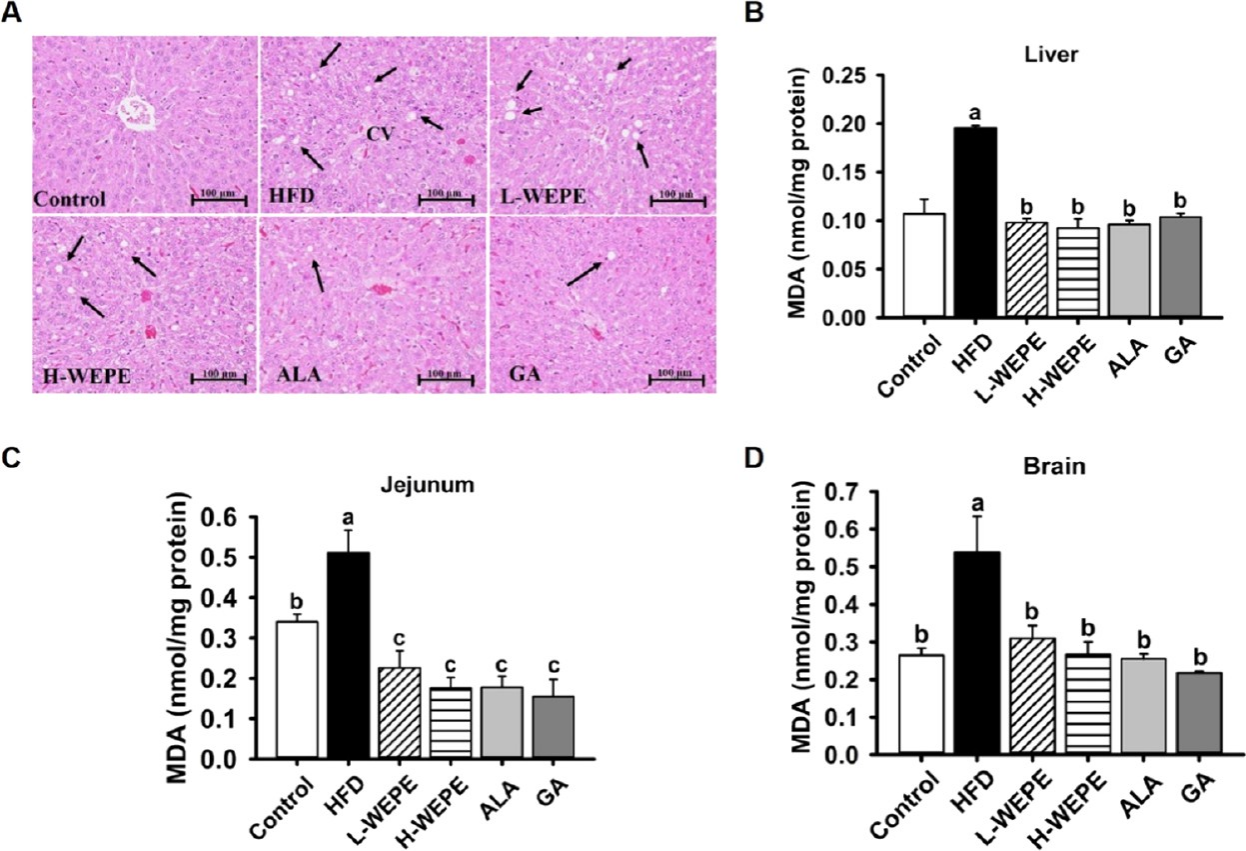
Effect of WEPE administration on (A) hepatic lipid accumulation and (B–D) levels of MDA in the liver, jejunum, and brain of HFD-fed rats. Arrow: fat vesicles; CV: central vein. Values with different letters in each column are significantly different (*p* < 0.05, *n* = 5).

Additionally, hepatic lipid accumulation was effectively induced in the HFD group but was inhibited by supplementation with WEPE, ALA, or GA in the HFD-treated group (Figure 3A). Furthermore, higher scores of macrovesicular and microvesicular steatosis were exhibited in the HFD group, which were significantly reversed in the L-WEPE, H-WEPE, ALA, and GA groups (Table 1). Consistently, the serum biochemical parameters TG and LDL-C were significantly increased in the HFD group, whereas the administration of WEPE, ALA, or GA effectively attenuated the accumulation of TG and LDL-C (Table 2).

**Table 1:** Effect of WEPE Administration on Hepatic Pathology in HFD-Induced SD Rats†

	histopathological finding		
group	fatty liver (general degree)	microvesicular steatosis	macrovesicular steatosis
control	0.0 ± 0.0^d^	0.0 ± 0.0^c^	0.0 ± 0.0^d^
HFD	2.0 ± 0.3^a^	2.0 ± 0.3^a^	1.8 ± 0.2^a^
L-WEPE	1.2 ± 0.4^b^	1.0 ± 0.0^b^	1.0 ± 0.3^b^
H-WEPE	1.0 ± 0.3^bc^	0.8 ± 0.2^b^	0.8 ± 0.2^bc^
ALA	0.4 ± 0.2^bcd^	0.2 ± 0.2^c^	0.2 ± 0.2^cd^
GA	0.2 ± 0.2^cd^	0.0 ± 0.0^c^	0.2 ± 0.2^cd^

† Values with different letters in each column are significantly different (*p* < 0.05, *n* = 5). Macrovesicular and microvesicular steatosis were scored based on the percentage of the total area using the following categories: 0 = normal; 1 = slight (<10%); 2 = moderate (10–33%); 3 = moderate/severe (33–66%); and 4 = severe/high (66–100%).

**Table 2:** Effect of WEPE Administration on Serum Biochemical Parameters in HFD-Induced SD Rats†

	biochemical parameters			
group	HDL-C (mg/dL)	CHOL (mg/dL)	TG (mg/dL)	LDL-C (mg/dL)
control	19.8 ± 1.1^a^	61.6 ± 6.3^ab^	38.8 ± 1.8^d^	6.5 ± 0.5^c^
HFD	19.2 ± 1.0^a^	68.0 ± 4.0^a^	134.6 ± 8.2^a^	10.5 ± 0.6^a^
L-WEPE	19.4 ± 0.8^a^	61.4 ± 2.0^ab^	111.0 ± 6.4^b^	8.2 ± 0.6^b^
H-WEPE	17.2 ± 1.6^a^	49.6 ± 3.2^c^	102.0 ± 4.9^b^	6.7 ± 0.6^c^
ALA	18.2 ± 1.3^a^	52.8 ± 2.6^bc^	60.4 ± 2.3^c^	6.4 ± 0.5^c^
GA	21.0 ± 1.0^a^	59.2 ± 2.1^abc^	47.2 ± 3.0^cd^	7.5 ± 0.3^bc^

† Values with different letters in each column are significantly different (*p* < 0.05, *n* = 5).

In addition, the mechanism by which obesity triggers oxidative stress and inflammation is well-known.^21^ In this study, the level of MDA was increased in the jejunum, brain, and liver tissues of the HFD group but was dramatically decreased in HFD-fed rats subjected to WEPE, ALA, and GA treatment (Figure 3B–D). Moreover, the secretion of proinflammatory cytokines (IL-1β, IL-6, and TNF-α) in the jejunum, brain, and liver tissues was significantly increased by HFD stimulation but was markedly decreased in the H-WEPE, ALA, and GA groups (Table 3).

**Table 3:** Effect of WEPE on the Levels of Liver Proinflammatory Cytokines in HFD-Induced SD Rats†

		group					
organ	item	control	HFD	L-WEPE	H-WEPE	ALA	GA
jejunum	IL-1β (ng/mg)	217.2 ± 23.7^c^	335.9 ± 17.6^a^	290.2 ± 19.8^ab^	273.0 ± 17.4^bc^	246.0 ± 12.7^bc^	233.7 ± 21.3^bc^
	IL-6 (ng/mg)	2.1 ± 0.3^b^	3.7 ± 0.3^a^	2.5 ± 0.3^b^	2.2 ± 0.1^b^	2.0 ± 0.2^b^	2.3 ± 0.3^b^
	TNF-α (pg/mg)	76.4 ± 8.1^b^	117.0 ± 19.2^a^	92.4 ± 9.0^ab^	84.3 ± 9.8^ab^	80.0 ± 11.8^b^	68.5 ± 6.4^b^
brain	IL-1β (ng/mg)	107.2 ± 8.9^d^	315.1 ± 18.5^a^	232.5 ± 12.3^b^	166.0 ± 18.1^cd^	214.9 ± 17.3^bc^	203.0 ± 35.3^bc^
	IL-6 (ng/mg)	0.2 ± 0.1^b^	0.4 ± 0.0^a^	0.2 ± 0.0^b^	0.2 ± 0.0^b^	0.2 ± 0.0^b^	0.2 ± 0.0^b^
	TNF-α (pg/mg)	80.4 ± 11.1^b^	155.9 ± 23.1^a^	109.2 ± 14.2^b^	95.6 ± 8.2^b^	94.2 ± 6.9^b^	92.3 ± 9.7^b^
liver	IL-1β (ng/mg)	0.6 ± 0.1^c^	1.3 ± 0.2^a^	1.0 ± 0.1^b^	0.6 ± 0.0^c^	0.6 ± 0.1^c^	0.6 ± 0.1^c^
	IL-6 (ng/mg)	2.3 ± 0.5^bc^	4.6 ± 0.7^a^	3.2 ± 0.6^b^	1.5 ± 0.2^c^	1.6 ± 0.1^c^	1.8 ± 0.2^c^
	TNF-α (pg/mg)	557.0 ± 54.7^c^	955.0 ± 40.5^a^	801.7 ± 12.0^b^	599.8 ± 75.1^c^	582.0 ± 15.8^c^	550.6 ± 39.3^c^

† Values with different letters in each column are significantly different (*p* < 0.05, *n* = 5).

### 3.3 WEPE Regulated Appetite-Related Genes and Fecal Lipid Excretion through Improving MG-Initiated Leptin Resistance

**Figure 4:**
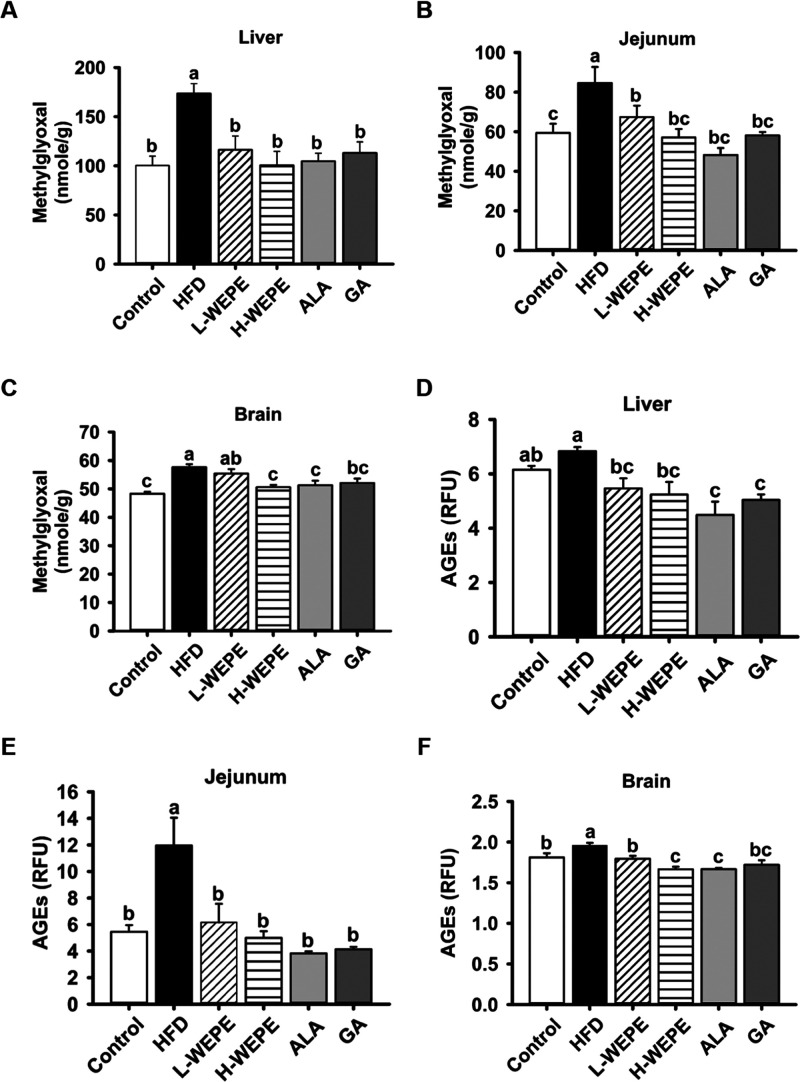
Effect of WEPE administration on the levels of tissue MG and AGEs. The levels of (A–C) MG and (D–F) AGEs in the liver, jejunum, and brain of HFD-fed rats. Values with different letters in each column are significantly different (*p* < 0.05, *n* = 5).

**Figure 5:**
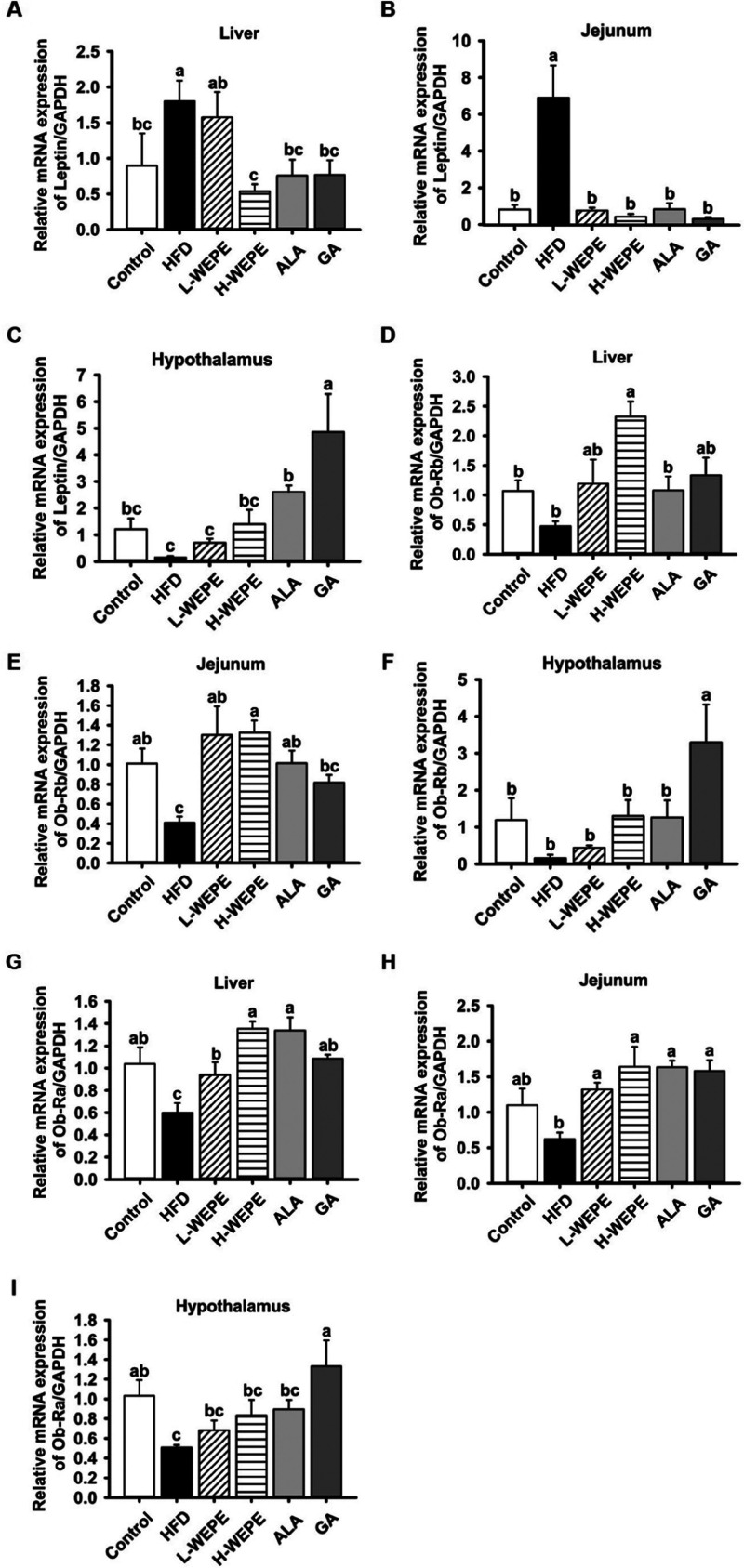
Effect of WEPE administration on the gene expression of leptin and leptin receptors in HFD-induced SD rats. The expression of (A–C) leptin, (D–F) Ob-Rb, and (G–I) Ob-Ra in liver, jejunum, and hypothalamus tissues of HFD-fed rats. Values with different letters in each column are significantly different (*p* < 0.05, *n* = 5).

Our previous evidence has shown that leptin resistance is evoked by MG-glycated leptin *in vitro*, which impairs lipid metabolism in FFA-treated HepG2 cells.^6^ Here, an animal study was performed to validate the *in vitro* data. The levels of MG in the jejunum, brain, and liver tissues were significantly elevated in the group receiving only the HFD compared with those in the control group (Figure 4A–C). Administration of WEPE, ALA, or GA effectively decreased HFD-induced MG accumulation (Figure 4A–C). Similar patterns of AGE levels were observed in jejunum, brain, and liver tissues (Figure 4D–F). Interestingly, a typical feature of leptin resistance was found that a high expression of the leptin gene in the jejunum and liver tissues was accompanied by a low leptin expression in the hypothalamus in the HFD group (Figure 5A–C). This phenomenon was ameliorated by WEPE, ALA, and GA intake compared with that in the HFD group (Figure 5A–C). The gene expression of leptin receptors (Ob-Ra and Ob-Rb) was reduced in the jejunum, hypothalamus, and liver tissues of the HFD group, whereas it was restored by WEPE, ALA, and GA supplementation (Figure 5D–I).

**Figure 6:**
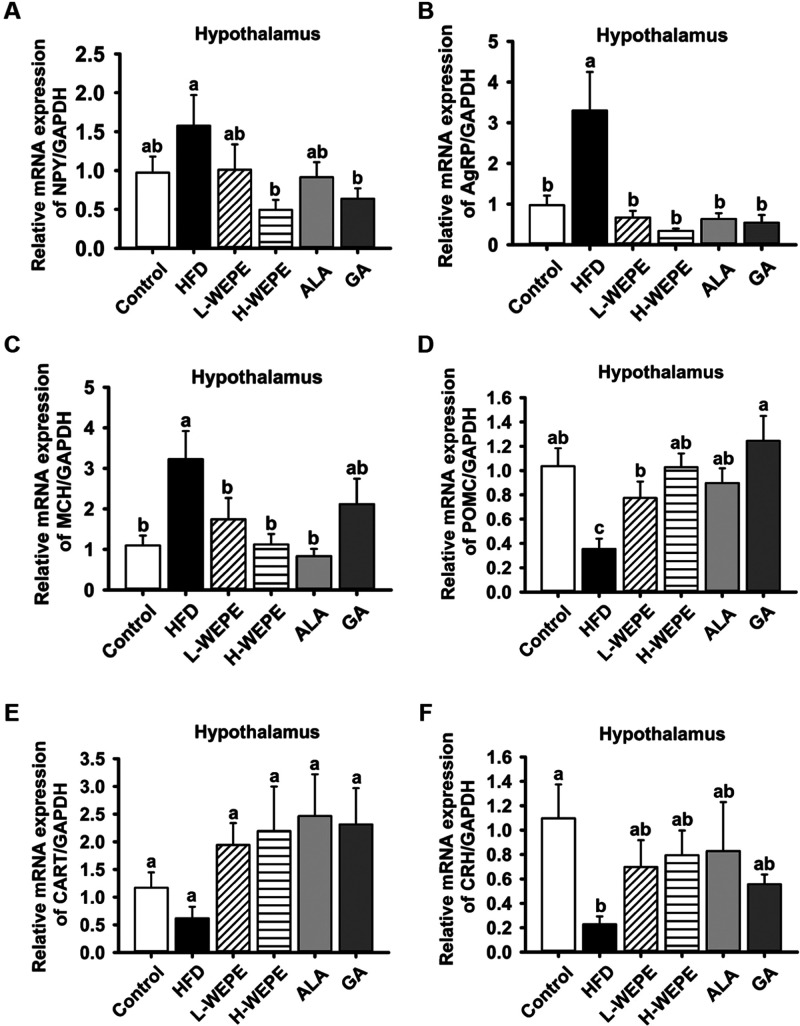
Effect of WEPE administration on the gene expression of orexigenic and anorexigenic neuropeptides in the hypothalamic tissue of HFD-induced SD rats. The expression of (A–C) orexigenic neuropeptides (NPY, AgRP, and MCH) and (D–F) anorexigenic neuropeptides (POMC, CART, and CRH). Values with different letters in each column are significantly different (*p* < 0.05, *n* = 5).

Leptin directly controls appetite by regulating the appetite-associated neuropeptides. Thus, the expression of orexigenic neuropeptides and anorexigenic neuropeptides in hypothalamic tissue was evaluated. As shown in Figure 6A–C, the gene expression of orexigenic neuropeptides (NPY, AgRP, and MCH) was increased in the HFD group and was prominently decreased by WEPE, ALA, and GA treatment, particularly AgRP gene expression. Concomitantly, the levels of anorexigenic neuropeptides (POMC, CART, and CRH) were reduced in the HFD group but were dramatically reversed in the WEPE, ALA, and GA groups, especially for POMC gene expression (Figure 6D–F).

**Figure 7:**
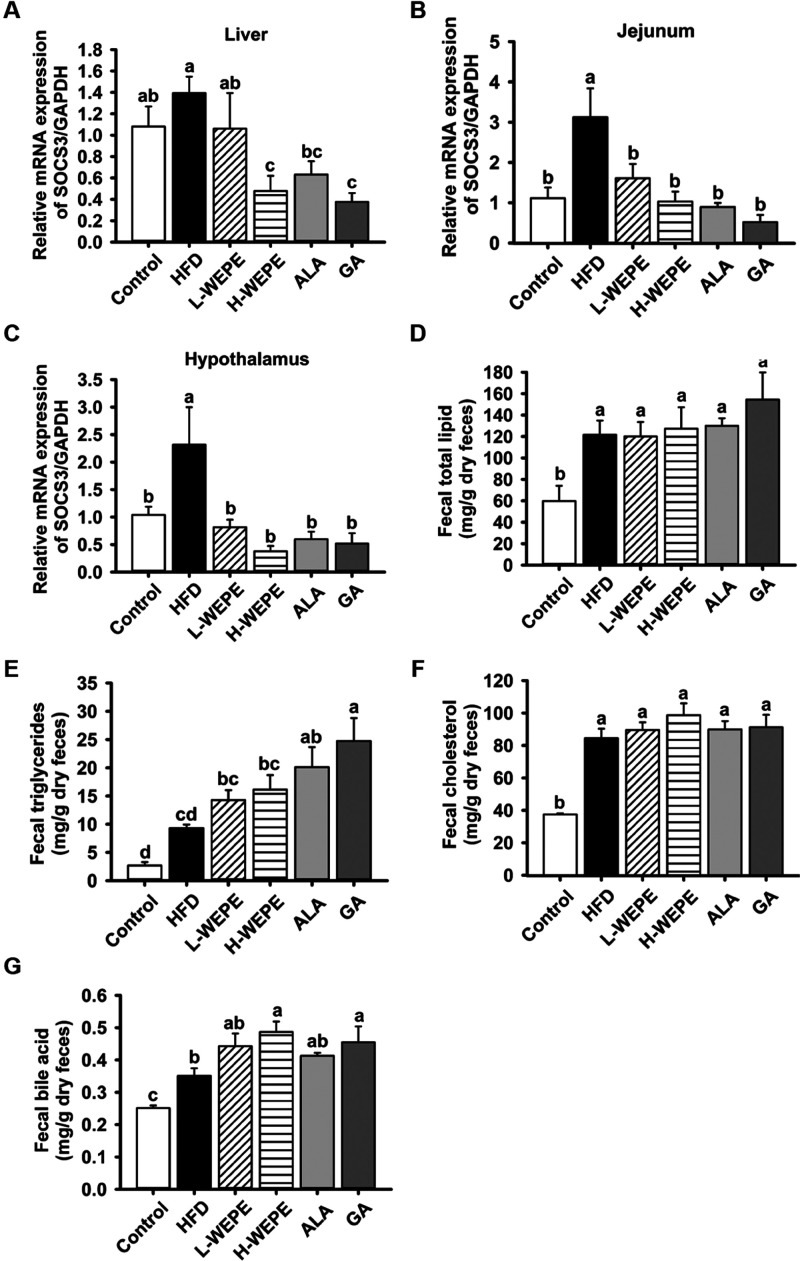
Effect of WEPE administration on the expression of SOCS3 and fecal lipid excretion in HFD-induced SD rats. The expression of SOCS3 in (A) liver, (B) jejunum, and (C) hypothalamus. The levels of fecal (D) total lipids, (E) TG, (F) TC, and (G) TBA in HFD-induced SD rats. Values with different letters in each column are significantly different (*p* < 0.05, *n* = 5).

Suppressor of cytokine signaling 3 (SOCS3) serves as the primary negative regulator of leptin-initiated signaling pathways involved in lipid metabolism. In addition, our previous evidence demonstrated that SOCS3 blocks leptin function and induces lipid accumulation *in vitro*.^6^ Here, high expression of the SOCS3 gene in the jejunum, hypothalamus, and liver was observed in the HFD group, which was significantly repressed in the H-WEPE, ALA, and GA groups (Figure 7A–C), indicating that lipid metabolism was accelerated by WEPE, ALA, and GA treatment.

Next, fecal lipid excretion, including total lipids, TG, TC, and TBA, was examined. Compared to those in the control group, significantly greater total lipid, TG, total cholesterol (TC), and total bile acid (TBA) excretion were found in the HFD group due to excessive consumption of fat (Figure 7D–G). Administration of WEPE, ALA, or GA significantly increased TG excretion in feces (Figure 7E), indicating that WEPE and its major compound GA potentially regulate appetite, lipid metabolism, and excretion by enhancing leptin sensitivity.

### 3.4 WEPE Manipulates the Gut Microbiota and Enhances SCFA Production to Maintain Intestinal Homeostasis

**Figure 8:**
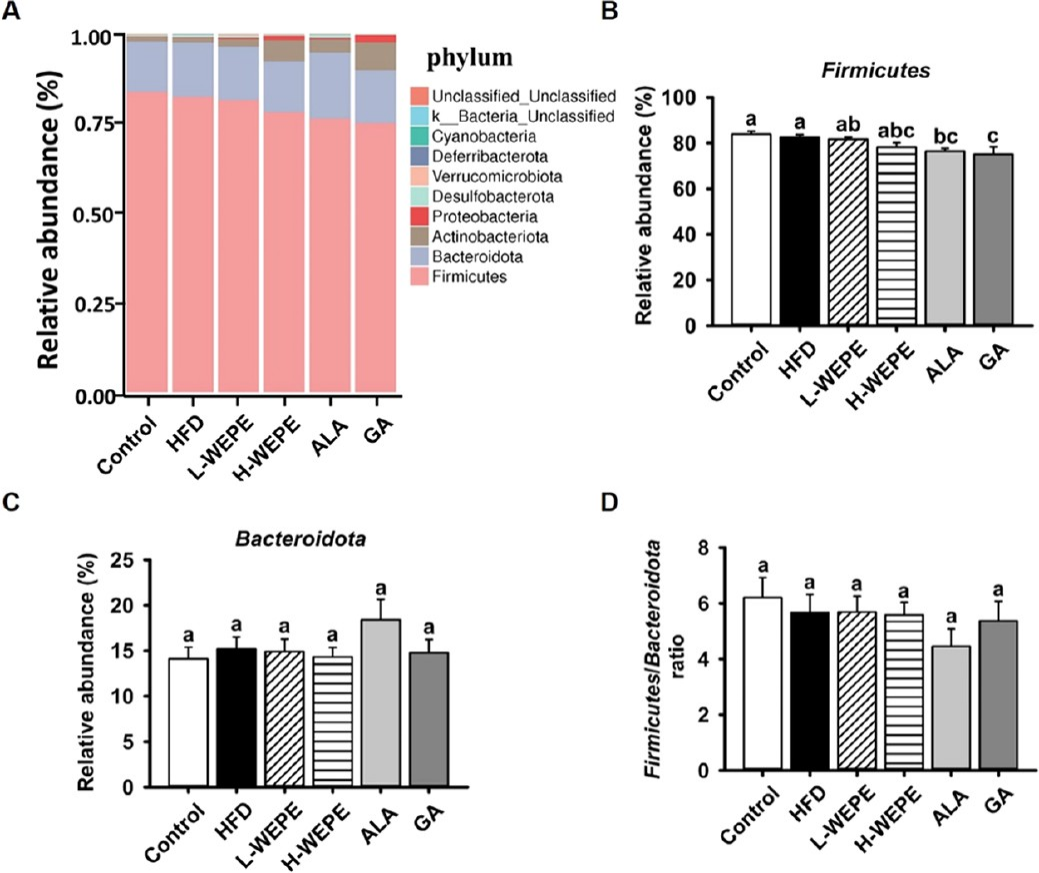
Effect of WEPE administration on the microbiota composition at the phylum level in HFD-induced SD rats. (A) Microbiota composition at the phylum level. (B) *Firmicutes*. (C) *Bacteroidota*. (D) F/B ratio.

**Figure 9:**
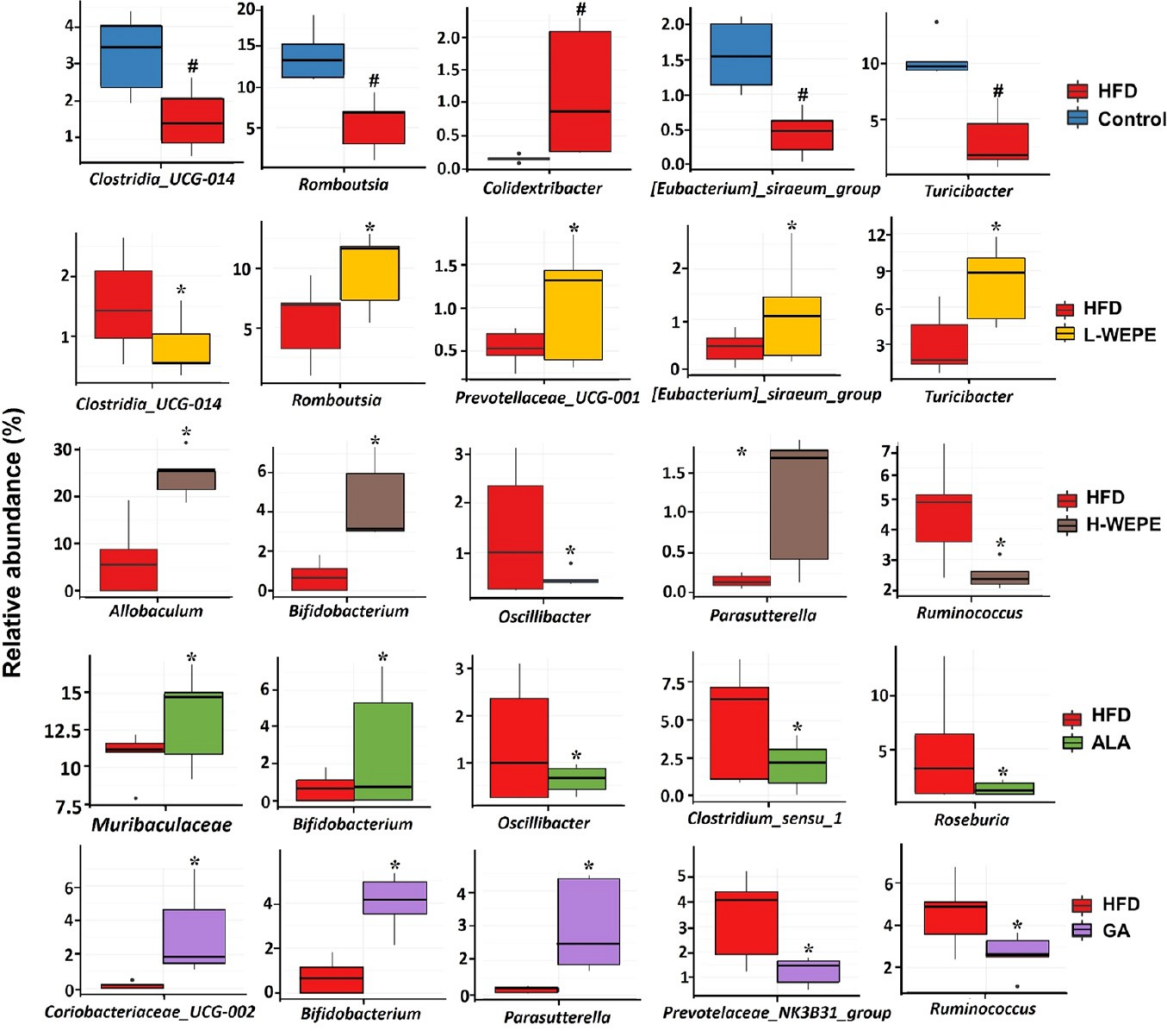
Effect of WEPE administration on the alteration of the microbiota composition in HFD-induced SD rats. Relative abundance of the top five genera of the gut microbiota in the groups, namely, the control versus HFD, HFD versus L-WEPE, HFD versus H-WEPE, HFD versus ALA, and HFD versus GA groups. Significant differences between means were analyzed by Student’s *t*-test (*n* = 5). #*p* < 0.05 versus the control group. *p < 0.05 versus the HFD group.

Beneficial bacteria play a vital role in SCFA production, which directly maintains intestinal homeostasis.^22,23^ Notably, the SCFA content was significantly lower in the HFD group than in the control group, and these changes were dramatically reversed by WEPE, ALA, and GA treatments (Table 4). Although the *Firmicutes*/*Bacteroidetes* ratio was not effectively altered (Figure 8A–D), the relative abundances of some beneficial bacteria, such as *Romoutsia*, *Turicibacter*, *Allobaculum*, *Bifidobacterium*, *Coriobacteriaceae_UCG-002*, and *Parasutterella*, were dominant in the WEPE, ALA, and GA groups (Figure 9).

**Table 4:** Effect of WEPE on the Levels of Fecal SCFAs in HFD-Induced SD Rats†

group	acetic acid	propionic acid	butyric acid	total SCFAs
control	27.0 ± 2.0^a^	6.4 ± 1.3^a^	17.7 ± 2.7^a^	51.1 ± 5.2^a^
HFD	16.5 ± 2.0^b^	1.2 ± 0.5^b^	5.9 ± 1.0^c^	23.6 ± 3.4^c^
L-WEPE	25.9 ± 0.9^a^	4.5 ± 0.8^a^	10.5 ± 1.0^bc^	40.8 ± 2.1^b^
H-WEPE	23.0 ± 1.6^a^	4.6 ± 0.5^a^	11.1 ± 1.3^b^	38.8 ± 2.3^b^
ALA	27.5 ± 2.0^a^	4.3 ± 1.0^a^	8.5 ± 1.3^bc^	40.3 ± 0.3^b^
GA	25.3 ± 3.2^a^	5.2 ± 1.4^a^	11.0 ± 1.1^b^	41.6 ± 3.8^ab^

† Values with different letters in each column are significantly different (*p* < 0.05, *n* = 5).

**Figure 10:**
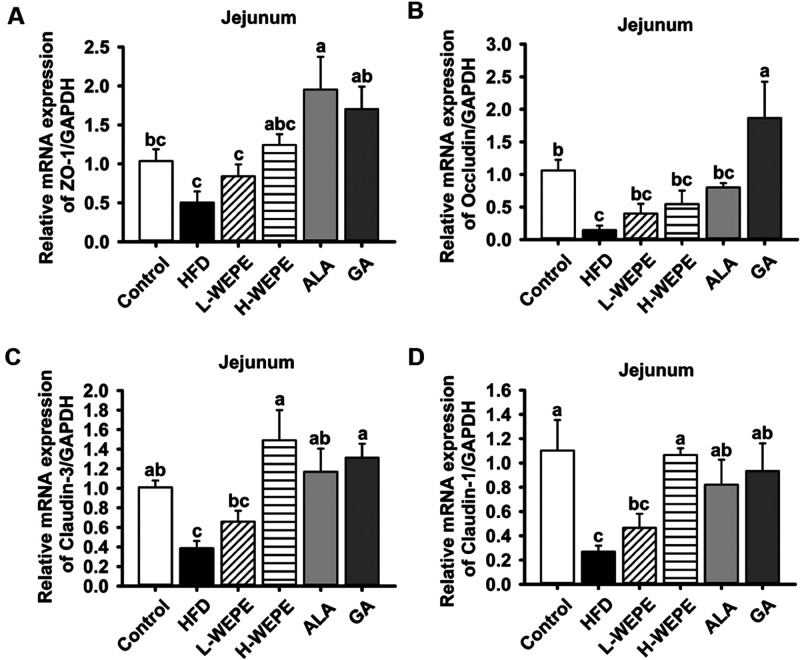
Effect of WEPE administration on the gene expression of tight junction proteins in HFD-induced SD rats. The levels of (A) ZO-1, (B) occludin, (C) claudin-3, and (D) claudin-1 in jejunum tissue. Values with different letters in each column are significantly different (*p* < 0.05, *n* = 5).

**Figure 11:**
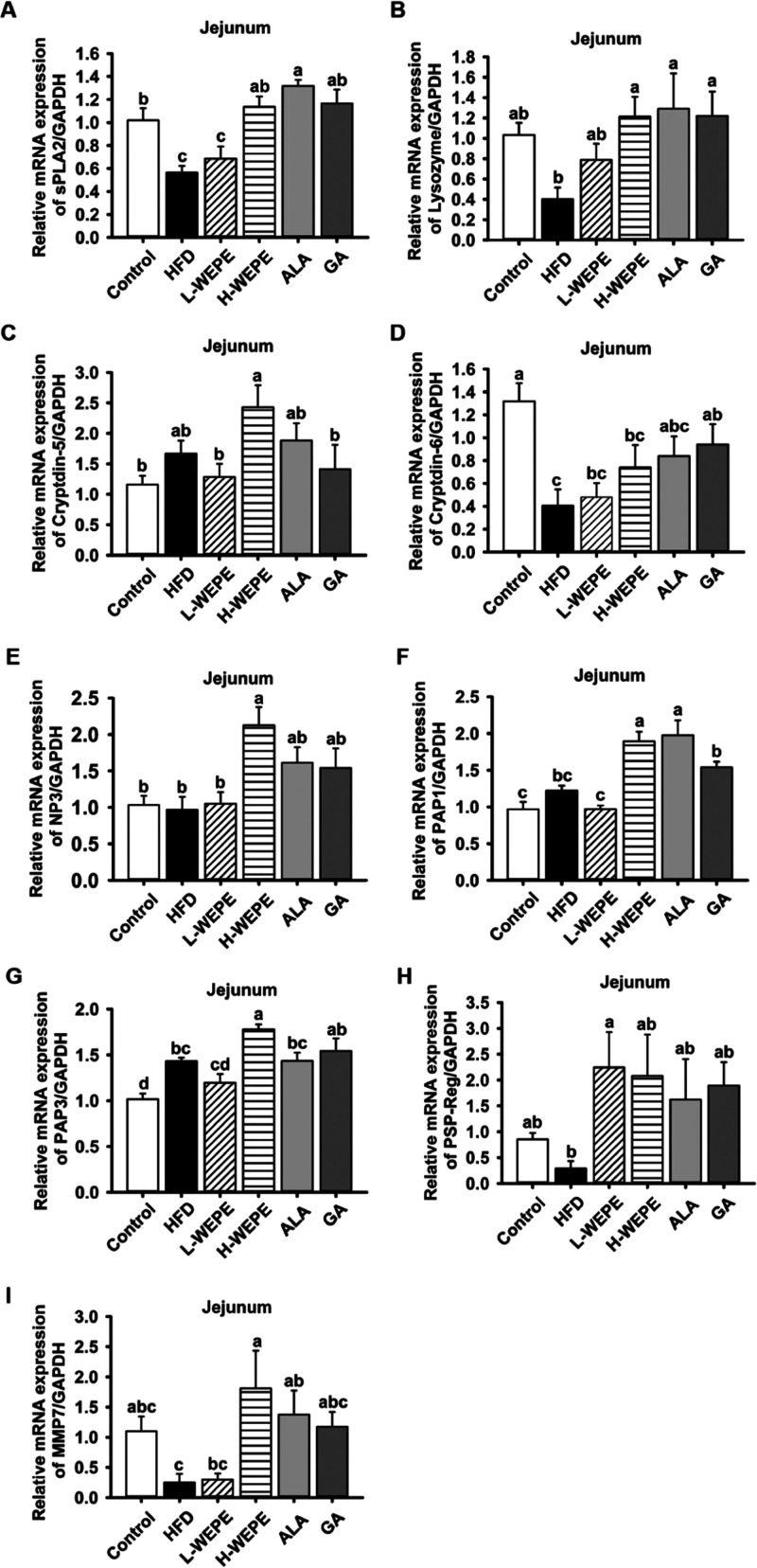
Effect of WEPE administration on the gene expression of antimicrobial peptides in HFD-induced SD rats. The levels of (A) sPLA2, (B) lysozyme, (C) cryptdin-5, (D) cryptdin-6, (E) NP3, (F) PAP1, (G) PAP3, (H) PSP/Reg, and (I) MMP-7 in jejunum tissue. Values with different letters in each column are significantly different (*p* < 0.05, *n* = 5).

Moreover, tight junctions and antimicrobial peptides are two critical factors for maintaining intestinal homeostasis.^24^ Here, the mRNA expression of tight junction proteins (ZO-1, occludin, claudin-3, and claudin-1) in the jejunum tissue was inhibited by HFD administration and was strongly enhanced by H-WEPE, ALA, and GA treatment (Figure 10A–D). Furthermore, the mRNA expression of antimicrobial peptides, such as sPLA2, lysozyme, cryptdin-5, cryptdin-6, NP3, PAP1, PAP3, PSP/Reg, and MMP-7, in jejunum tissue was evaluated. The gene expression of sPLA2, lysozyme, cryptdin-6, PSP/Reg, and MMP-7 was significantly inhibited in the HFD group but restored by WEPE, ALA, or GA treatment (Figure 11A–I). Although the gene expression of NP3 and PAP1 was not significantly different in the HFD group compared to that in the control group, supplementation with WEPE and ALA effectively increased the level of NP3 and PAP1 gene expression in HFD-fed rats (Figure 10E,F). The above evidence showed that WEPE improved intestinal homeostasis through regulating tight junctions and antimicrobial peptides.

### 3.5 Improving Leptin Resistance, Reducing Appetite, and Maintaining Intestinal Homeostasis via the Gut Microbiota–Brain Axis in HFD-Treated Rats

**Figure 12:**
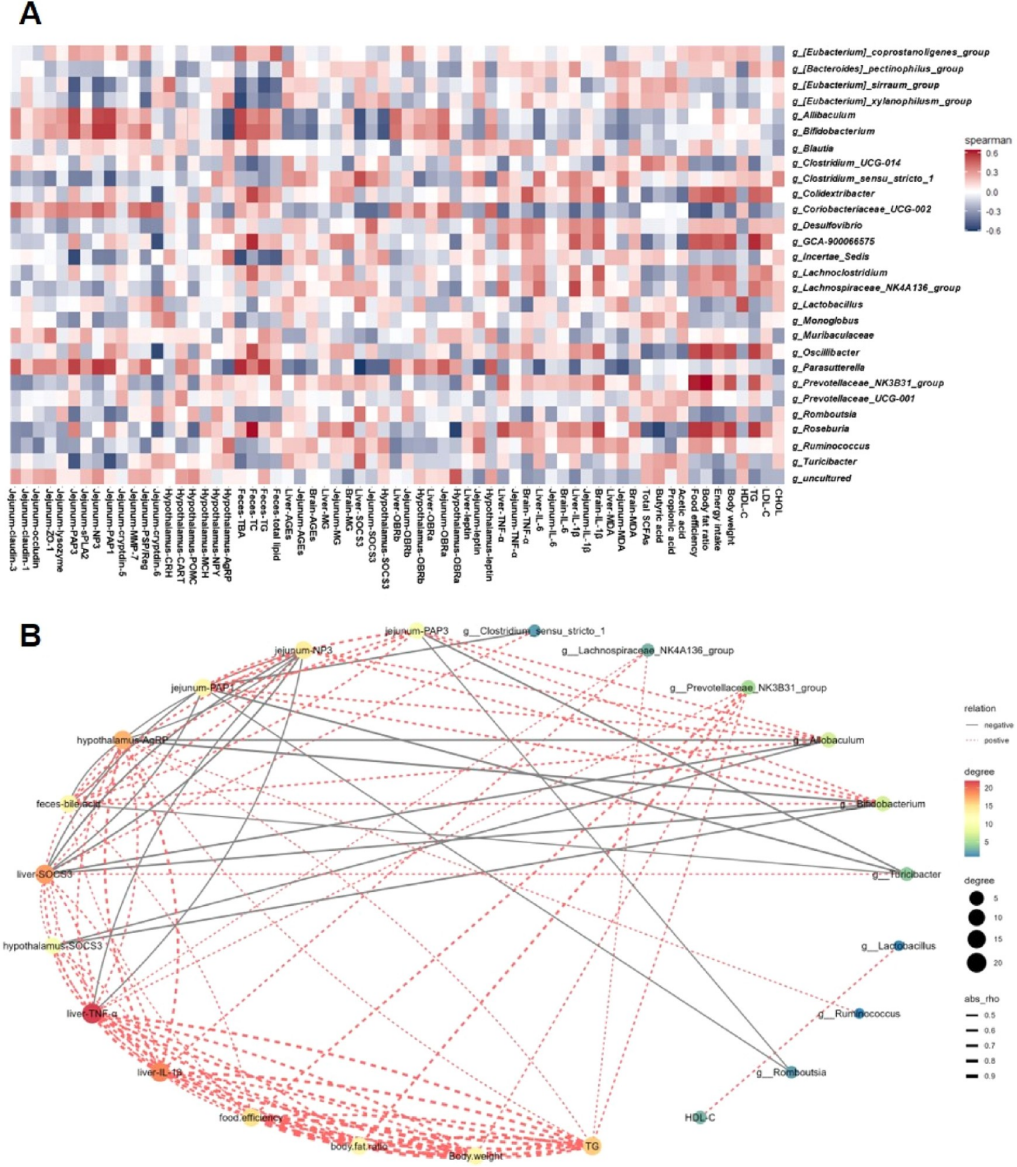
Relationships between the biochemical parameters and the gut microbiota composition. (A) Heatmap of Spearman’s correlation. Red represents a positive correlation, and blue represents a negative correlation. (B) Network construction relating bacterial species and biochemical parameters. The gray solid line represents a negative correlation, and the red dotted line represents a positive correlation. The size and color of the circles are proportional to the correlation strength.

Previous evidence has shown that the expression of antimicrobial peptides and tight junction proteins is increased in leptin-treated mice to ameliorate colitis,^25^ indicating that WEPE may improve intestinal homeostasis by suppressing leptin resistance via the gut microbiota–brain–liver axis. As shown in Figure 12A, the correlation between all of the results and the gut microbiota composition was evaluated through Spearman correlation analysis. The study revealed positive correlations between the relative abundances of *Roseburia*, *Colidexribacter*, and *Oscillibacter* and various physiological parameters (body weight, body fat ratio, energy intake, food efficiency ratio, TG, and proinflammatory cytokines) but negative correlations with SCFA production. Moreover, a positive correlation between bacteria (*Lachnospiraceae_NK4A136-group* and *Ruminococcus*) and obesity-related indicators (MG, AGEs, MDA, orexigenic neuropeptides, jejunum leptin, and liver leptin) was observed in HFD-fed rats. In contrast, *Lachnospiraceae_NK4A136-group* and *Ruminococcus* presented negative correlations with cryptdin-6, tight junction proteins, Ob-Ra, Ob-Rb, and anorexigenic neuropeptides.

Interestingly, *Allobaculum*, *Bifidobacterium*, *Parasutterella*, *Romboutsia*, and *Coriobacteriaceae_UCG-002* were found to be beneficial for improving leptin resistance and intestinal homeostasis via the gut–brain–liver axis. The relative abundances of *Allobaculum*, *Bifidobacterium*, and *Parasutterella* were positively correlated with fecal lipid excretion, leptin receptors, tight junctions, and antimicrobial peptides but negatively correlated with MDA, jejunum leptin, liver leptin, SOCS3, and orexigenic neuropeptides (Figure 12A). Furthermore, *Romboutsia* was positively correlated with SCFAs and anorexigenic neuropeptides but negatively correlated with physiological parameters (body weight, body fat ratio, energy intake, and food efficiency ratio) (Figure 12A). Additionally, *Coriobacteriaceae_UCG-002* was positively correlated with the leptin receptor (Ob-Ra, Ob-Rb), tight junction proteins, and antimicrobial peptides, while its physiological parameters (body weight, body fat ratio, energy intake, and food efficiency ratio), proinflammatory cytokines, MG, AGEs, jejunum leptin, liver leptin, SOCS3, and orexigenic neuropeptides were negatively correlated with *Coriobacteriaceae_UCG-002* (Figure 12A).

Moreover, network construction produced similar results: *Rumonococcus*, *Lachnospiraceae_NK4A136-group*, *Prevotellaceae_NK3B31_group*, and *Clostridium_sensu_stricto_1* were involved in the accumulation of IL-1β, TNF-α, MG, AGEs, TG, AgRP, and SOCS3 for the development of obesity (Figure 12B). In contrast, *Allobaculum*, *Bifidobacterium*, and *Lactobacillus* were positively associated with Ob-Ra, Ob-Rb, antimicrobial peptides (PAP1, NP3, and PAP1), tight junction proteins, and HDL-C (Figure 12B). The above evidence indicated that WEPE improved leptin resistance, reduced appetite, and maintained intestinal homeostasis via the gut microbiota–brain axis in HFD-administered rats.

## 4 Discussion

Obesity is a significant global health challenge that profoundly affects both individuals and society as a whole. It poses substantial health risks and contributes to a significant societal burden. Leptin resistance and microbiota variation are involved in the progression of obesity.^26^ Leptin is not only involved in appetite control but also maintains intestinal homeostasis in dextran sulfate sodium (DSS)-induced colitis mice via the regulation of antimicrobial peptides and tight junction proteins.^25,27^ Additionally, a systematic review revealed that probiotic and synbiotic supplementation significantly decreases the serum/plasma concentrations of leptin and appetite in patients with nonalcoholic fatty liver disease (NAFLD, *n* = 1536).^27^ The above evidence indicates that leptin acts as a key factor in manipulating appetite and intestinal homeostasis via microbiota–gut–brain–liver interactions; however, whether this phenomenon occurs in individuals with obesity-evoked leptin resistance is still unclear. The underlying mechanism was revealed in this work by MG-triggered leptin resistance through the gut–brain–liver axis in HFD-fed rats, whereas this resistance was relieved by WEPE and GA administration.

To evaluate the novelty of our findings, only 12 studies were found in PubMed by searching the keywords “*P. emblica*” and “obesity”. Our previous evidence demonstrated that WEPE has powerful potential for attenuating lipid accumulation and obesity-derived diseases such as nonalcoholic steatohepatitis and cognitive decline through obstacles to MG-induced leptin or insulin resistance.^5,6,28,29^ Furthermore, we hypothesized that WEPE might contribute to appetite control and intestinal homeostasis maintenance through the leptin-regulated gut–brain–liver axis. Consistent with this possibility, MG accumulation and leptin resistance were observed in the jejunum, brain, and liver and promoted appetite and dysbiosis through the gut–brain–liver axis in HFD-induced rats, whereas WEPE and its major bioactive compound GA effectively enhanced the expression of anorexigenic neuropeptides, tight junction genes, and antimicrobial peptides by altering the microbiota composition; these effects were comparable to those of ALA treatment.

Leptin is an important hormone that is involved in regulating appetite, food intake, and lipid metabolism.^30^ In this study, MG accumulation-induced leptin resistance subsequently induced an increase in the level of orexigenic neuropeptides (NPY, AgRP, and MCH) in the HFD group, whereas WEPE administration effectively decreased the level of expression of orexigenic neuropeptides and upregulated the level of expression of anorexigenic neuropeptides (POMC, CART, and CRH), indicating that WEPE attenuated MG-associated leptin resistance in HFD-treated rats. Although WEPE enhanced the gene expression of anorexigenic neuropeptides, body weight was reduced without an energy intake. Previous evidence has shown that leptin administration decreases body weight and fat intake by increasing lipid dissipation but fails to reduce food intake.^31,32^ Moreover, the inhibition of POMC did not significantly affect food intake,^33^ indicating that controlling food intake is complex. Nevertheless, the levels of serum CHOL, TG, and LDL-C were significantly reduced by WEPE treatment in HFD-fed rats. Moreover, a higher excretion of fecal TG and bile acid was also observed in the H-WEPE, ALA, and GA groups than in the HFD group, indicating that WEPE may increase body weight and fat accumulation through lipid excretion.

MG and MG-derived AGEs are notorious for their ability to facilitate the treatment of various diseases, including diabetes, cardiovascular disease, obesity, Alzheimer’s disease, cancer, and age-related diseases.^34^ Until now, the exact relationship between MG and leptin resistance has remained unclear. Currently, our study demonstrated that MG-glycated leptin causes leptin resistance, ROS production, inflammatory cytokine secretion, and lipid accumulation in FFA-treated HepG2 cells.^6^ Here, an animal study supported the cell culture data and showed that MG and AGE accumulation in jejunum, brain, and liver tissues occurred concomitantly with leptin resistance, SOCS3 expression, MDA accumulation, and proinflammatory cytokine (IL-1β, IL-6, and TNF-α) accumulation in the jejunum, brain, and liver tissues of HFD-fed rats. Notably, supplementation with WEPE and GA effectively reduced the accumulation of MG, and AGEs subsequently restored leptin functions, which was not only found in our previous *in vitro* study^6^ but also evidenced in this *in vivo* study.

A previous report showed that *Panax notoginseng* saponins increase *A. muciniphila* and *Parabacteroides distasonis* abundances and result in the enhancement of energy expenditure by activating the leptin-AMPK/STAT3 signaling pathway in HFD-induced obese wild-type mice but fail to reach these effects in leptin gene-deficient mice.^35^ Moreover, the administration of leptin promotes antimicrobial peptide and tight junction protein expression to ameliorate colitis via gut microbiota modulation, indicating that leptin directly influences gut microbiota modulation.^25^

In the present study, the WEPE, ALA, and GA groups not only exhibited increased leptin expression but also increased microbiota abundance. Notably, supplementation with WEPE, ALA, or GA increased SCFA levels and the abundance of SCFA-producing bacteria (including *Romboutsia* and *Turicibacter*), which strongly negatively correlated with body weight.^36,37^ Additionally, HFD feeding has been reported to decrease the relative abundance of *Allobaculum*, *Coriobacteriaceae_UCG-002*, and *Parasutterella*, which are negatively related to liver and serum lipid levels.^38^ Intriguingly, *Bifidobacterium* species play various health-beneficial roles, such as alleviating the inflammatory response and regulating tight junctions,^39^ maintaining intestinal homeostasis,^40^ combating obesity via lipid metabolism,^41^ and producing antimicrobial peptides against *Helicobacter pylori*.^42^ In addition, a clinical trial showed that 8 weeks of exercise training significantly improved insulin sensitivity and obesity by reducing the abundance of *Ruminococcus*.^43^ Moreover, *Ruminococcus*, *Oscillibacter*, and *Colidexibacter* are strongly associated with inflammatory and oxidative stress during the progression of obesity.^38,44,45^

Consistent with these findings, this study provided compelling evidence that supplementation with WEPE, ALA, and GA increased leptin levels and the abundance of health-beneficial microbes (*Romboutsia*, *Turicibacter*, *Allobaculum*, *Bifidobacterium*, *Coriobacteriaceae_UCG-002*, and *Parasutterella*) and decreased the abundance of obesity-related bacteria (*Ruminococcus*, *Oscillibacter*, and *Colidexibacter*) concomitantly, which correlated with the process of obesity amelioration.

In summary, our results manifested that WEPE and GA restored MG-triggered leptin resistance, which consequently repressed MDA and proinflammatory cytokines (TNF-α, IL-1β, IL-6), orexigenic neuropeptides (NPY, AgRP, and MCH), steatosis, and lipid accumulation by enhancing the expression of anorexigenic neuropeptides (POMC, CART, and CRH), SCFAs (acetic acid, propionic acid, and butyric acid) production, TG excretion, tight junction (ZO-1, occluding, claudin-3, and claudin-1), antimicrobial peptides (sPLA2, lysozyme, cryptdin-5, cryptdin-6, NP3, PAP1, PAP3, PSP/Reg, and MMP-7), and health-beneficial bacteria (*Romboutsia*, *Turicibacter*, *Allobaculum*, *Bifidobacterium*, *Coriobacteriaceae_UCG-002*, and *Parasutterella*) against HFD-induced gut disorder through the gut–brain–liver axis.

## Supporting Information Available

Detailed primer sequences utilized for real-time PCR analysis (Table S1) (PDF)

## Abbreviations

**AGEs**: advanced glycation end products
**AgRP**: agouti-related protein
**ALA**: alagebrium chloride
**CART**: cocaine and amphetamine-regulated transcript
**CRH**: corticotropin-releasing hormone
**GA**: gallic acid
**MCH**: melanin-concentrating hormone
**MG**: methylglyoxal
**MMP-7**: matrix metalloproteinase-7
**NP3**: neutrophil peptide 3
**NPY**: neuropeptide Y
**PAP**: pancreatic-associated proteins
**POMC**: pro-opiomelanocortin
**PSP/Reg**: pancreatic secretory peptide/islet regenerating peptide
**SCFAs**: short-chain fatty acids
**SOCS3**: suppressor of cytokine signaling 3
**sPLA2**: secretory phospholipase A2
**WEPE**: water extract from *Phyllanthus emblica* L
**ZO-1**: zonula occluding-1
