# Supplementary material for: Phyllanthus emblica Fruit Improves Obesity by Reducing Appetite and Enhancing Mucosal Homeostasis via the Gut Microbiota–Brain–Liver Axis in HFD-Induced Leptin-Resistant Rats
Source: J Agric Food Chem. 2024 Apr 25;72(18):10406–19. doi: 10.1021/acs.jafc.4c01226 (PMC11082930; doi:10.1021/acs.jafc.4c01226)
Supplement: Supplementary file 1 — jf4c01226_si_001.pdf [file jf4c01226_si_001.pdf]

Phyllanthus emblica fruit improves obesity by reducing appetite and enhancing mucosal homeostasis via the gut microbiota-brain-liver axis in HFD-induced leptin-resistant rats

Hsin-Yu Chang[a,1], Sheng-Yi Chen[a,1], Jer-An Lin[b], Ying-Yin Chen[a], Ying-Ying Chen[a], Yu-Chen Liu[a], Gow-Chin Yen[a,*]

[a] Department of Food Science and Biotechnology, National Chung Hsing University, 145 Xingda Road, Taichung 40227, Taiwan

[b] Graduate Institute of Food Safety, National Chung Hsing University, 145 Xingda Road, Taichung 40227, Taiwan

Corresponding author.

E-mail address: gcyen@nchu.edu.tw (G.C. Yen).

[1] These authors contributed equally to this work.

Supporting information

Table S1. Primers used for real-time PCR

Gene	Sequences (5′-3′)
GAPDH	F: TGTGTCCGTCGTGGATCTGA<br>R: CCTGCTTCACCACCTTCTTGA
Leptin	F: GCGACTCTCAGGAAAAGGACT<br>R: GAGTTGTTGGTCTCTTCTGGGT
Ob-Rb	F: GCATGCAGAATCAGTGATATTTGG<br>R: CAAGCTGTATCGACACTGATTTCTTC
Ob-Ra	F: ACACTGTTAATTTCACACCAGAG<br>R: AGTCATTCAAACCATAGTTTAGG
SOCS3	F: GCCAGTGCCCCGCTTTGACT<br>R: GAGGAGGGTTCCGTCGGTGGT
NPY	F: TATCCCTGCTCGTGTGTTTG<br>R: GTTCTGGGGGCATTTTCTG
AgRP	F: AGCAGACCGAGCAGAAGATG<br>R: GACTCGTGCAGCCTTACACA
MCH	F: CCGCAGAAAGAT CGGTTGTT<br>R: TGGTCCTTTCAGAGCGAGGTA
POMC	F: ACCTCACCACGGAAAGCAA<br>R: CGGGGATTTTCAGTCAAGG
CART	F: GCCAAGTCCCCATGTGTGAC<br>R: CACCCCTTCACAAGCACTTCA
CRH	F: TGGATCTCACCTTCCACCTTCTG<br>R: CCGATAATCTCCATCAGTTTCCTG
Cryptdin-5	F: GACCAGGTTGTTTCTGTCTCCATTG<br>R: TGAGGCTTCCGTATCTCTTGTTGC
Cryptdin-6	F: AGCAACCATCAGATGAGGACCAGG<br>R: ACCTTGAGCACAGAACGCAGTGG
NP3	F: TTTGGAGGGGATAAAGGCACTGC<br>R: TCAGCAACAGAGTCGGTAGATGCG
PAP1	F: TGCCAGAAGAGACCTGAAGGACAC<br>R: TTGTTACTCCACTCCCATCCACCTC
PAP3	F: CCAAGAACCCAACAGAGGTGGATG<br>R: GGTCCCACAGTGACTTCCAGAGACAG
PSP/Reg	F: GTTTCTCTACAAATCCTGGGACACTGG

	R: TTGGGCATCACAACTGTTATCTCTCC
Lysozyme	F: ACACAAGCCAGAAACTACAACC
	R: TCAGACTCCGCAGTTCCGAAT
sPLA2	F: TGACTCATGACTGTTGTTACAACC
	R: TCTCAGGACCCTCTTAGGTACTA
MMP-7	F: CGGCGGAGATGCTCACTTTGAC
	R: TGGCTCAGGAAGGGCGTTTGC
ZO-1	F: AGTTCTGCCCTCAGCTACCA
	R: GCTTAAAGCTGGCAGTGTC
Occludin	F: ACAAAGAGCTCTCTCGTCTCG
	R: CATAGTCTCCCACCATCCTC
Claudin-3	F: ATTCATCGGCAGCAGCATC
	R: CCAGCAGCGAGTCGTACATC
Claudin-1	F: GGACAACATCGTGACTGCTCAGG
	R: TGCCAATTACCATCAAGGCTCTGG

F: forward primer; R: reverse primer.
